# Neuroendocrine-immune perturbations in metabolic disease: pathophysiological mechanisms underlying cognitive impairment

**DOI:** 10.3389/fnhum.2026.1857087

**Published:** 2026-07-13

**Authors:** Aviva Fraer, Sanaz Saleh, Dana Tasabehji, Durga Shankar Sharma, Mohamad Mokadem

**Affiliations:** 1 Department of Internal Medicine, Roy J. and Lucille A. Carver College of Medicine, University of Iowa, Iowa City, IA, United States; 2 Iowa Neuroscience Institute, Roy J. and Lucille A. Carver College of Medicine, University of Iowa, Iowa City, IA, United States; 3 Fraternal Order of Eagles Diabetes Research Center, University of Iowa, Iowa City, IA, United States; 4 Iowa City VA Health Care System, Iowa City, IA, United States

**Keywords:** adipokines, cognitive impairment, cytokines, hypothalamic inflammation, metabolic-associated steatotic liver disease, microgliosis, neurodegeneration, neuro–immune–endocrine axis

## Abstract

The escalating global prevalence of obesity and associated metabolic disorders has catalyzed intensive investigation into their multisystemic ramifications. Recent scientific investigations elucidate the sophisticated bidirectional relationship between systemic metabolic aberrations and central nervous system (CNS) dysfunction, orchestrated via complex neuro–immune–endocrine signaling cascades. This integrative analysis consolidates extant literature regarding the mechanisms by which adiposity, metabolic-associated steatotic liver disease (MASLD), and systemic metabolic perturbations disrupt neuro–immune–endocrine communication networks, consequently precipitating neuroinflammatory processes, cognitive dysfunction, and enhanced vulnerability to neurodegenerative disorders. We examine the molecular and cellular mechanisms underlying this pathophysiological cascade, including adipokine dysregulation, systemic inflammatory mediator elevation, hypothalamic inflammation, microglial activation, blood-brain barrier disruption, and subsequent neuronal dysfunction. Furthermore, we analyze the modulatory roles of key endocrine axes—including the hypothalamus, the pituitary and the adrenal glands-, and growth hormone/insulin-like growth factor systems—in metabolic-neural crosstalk. By elucidating these complex pathways, we aim to highlight mechanistically informed therapeutic targets and combinatorial strategies that may attenuate neuroinflammatory processes and cognitive deterioration in metabolically compromised individuals.

## 1 Method

This review was designed as a narrative, mechanism-focused synthesis of literature examining how obesity, metabolic-associated steatotic liver disease (MASLD), insulin resistance, and related metabolic disturbances influence neuro–immune–endocrine signaling and cognitive function. The central review question was to identify and integrate evidence linking peripheral metabolic dysfunction with central neuroinflammation, endocrine dysregulation, and neurological outcomes.

### 1.1 Search strategy and data sources

A structured literature search was performed in PubMed/MEDLINE, Scopus, and Google Scholar from database inception to March 31, 2026, with the final search conducted on March 31, 2026. Search terms were combined using Boolean operators and included: “obesity,” “metabolic-associated steatotic liver disease,” “MASLD,” “NAFLD,” “neuroinflammation,” “microglia,” “astrocytes,” “blood–brain barrier,” “hypothalamic inflammation,” “cognition,” “cognitive impairment,” “HPA axis,” “thyroid axis,” “growth hormone,” “IGF-1,” “adipokines,” “insulin resistance,” and “gut–liver–brain axis.” Searches emphasized peer-reviewed articles published in English.

### 1.2 Screening and study selection

Titles and abstracts were screened for relevance to three predefined domains: (1) peripheral metabolic and inflammatory mechanisms; (2) central nervous system (CNS) neuroinflammatory and neuroglial responses; and (3) endocrine and neuroendocrine pathways linking metabolic dysfunction to cognitive or neurobehavioral outcomes.

Full-text articles were retrieved when abstracts suggested direct relevance to at least one of these domains. Additional articles were identified through backward citation searching of key reviews and landmark primary studies.

### 1.3 Inclusion criteria

Articles were considered for inclusion if they met one or more of the following criteria:

1. Provided mechanistic insight into signaling pathways linking metabolic dysfunction to the brain (e.g., adipokines, cytokines, hepato-neural, or gut–liver–brain pathways).
2. Examined neuroinflammatory, neuroglial, or cognitive consequences of obesity, MASLD, insulin resistance, or related metabolic disturbances in humans or relevant animal models.
3. Evaluated alterations in neuroendocrine axes (e.g., HPA, HPT, GH/IGF-1) in the context of metabolic dysfunction, with direct implications for CNS structure or function.
4. Discussed therapeutic interventions targeting these pathways (metabolic, anti-inflammatory, or neuroendocrine) with mechanistic or cognitive/neuroimaging outcomes.

Within these criteria, priority was given to original studies, systematic reviews, meta-analyses, and high-impact mechanistic reviews that demonstrated strong mechanistic depth, translational relevance, and consistency with the conceptual framework of the review.

### 1.4 Exclusion criteria

Studies were excluded if they:

- Were not available in English.
- Lacked sufficient relevance to neuro–immune–endocrine interactions or cognitive outcomes (e.g., focused solely on peripheral metabolic endpoints without CNS measures or mechanisms).
- Focused exclusively on unrelated neurological or endocrine conditions without a clear metabolic component.
- Duplicated concepts are already covered by stronger, more recent, or more comprehensive sources.

Because this is a narrative, mechanism-focused review rather than a formal systematic review or meta-analysis, we did not perform pooled quantitative analyses or formal risk-of-bias assessments.

## 2 Introduction

The dramatic global rise in obesity and metabolic disorders represents one of the most significant public health challenges of the 21st century. Emerging evidence reveals a complex interplay between peripheral metabolic dysfunction and central nervous system (CNS) pathophysiology, mediated through intricate neuro–immune–endocrine networks that coordinate metabolic, inflammatory, and neural responses (Hotamisligil, 2006; Mitra et al., 2022; Le Thuc and García-Cáceres, 2024). Growing evidence suggests that a constellation of metabolic derangements induces a state of chronic neuro-inflammation, disrupting neuro–immune–endocrine signaling pathways and contributing to cognitive impairment and heightened susceptibility to neurodegenerative diseases (Mitra et al., 2022; Chu et al., 2020; O'Brien et al., 2017).

In obesity, adipokine profiles become dysregulated, and systemic inflammatory mediators rise throughout the body. In the brain, particularly in the hypothalamus, this increased inflammatory load activates microglia and triggers local neuroinflammation. These processes can also weaken the blood–brain barrier, facilitating the entry of peripheral inflammatory and metabolic signals into the CNS and ultimately promoting neuronal dysfunction (Guillemot-Legris and Muccioli, 2017; Tilg et al., 2025; Yamazaki and Kanekiyo, 2017). Those signaling cascades occur along the hypothalamic–pituitary–adrenal (HPA) as well as the hypothalamic–pituitary–thyroid (HPT) axes and involve a two-way neurohormonal crosstalk among several key regulatory organs (Boelen et al., 2011; McEwen, 2007). Dysregulation of these pathways alters neuro–immune–endocrine integration and may offer targets for future disease-modifying interventions (Livingston et al., 2020).

The World Health Organization estimates that over 890 million adults worldwide were obese in 2022, with prevalence projected to continue rising (World Health Organization, 2025). While the deleterious effects of obesity on cardiovascular and metabolic health have been extensively characterized, emerging evidence highlights the profound impact of metabolic dysfunction on neurological health and cognitive function (Le Thuc and García-Cáceres, 2024; Pugazhenthi et al., 2017; Opel et al., 2021). The brain, once considered an immunologically privileged organ isolated from peripheral perturbations, is now recognized to maintain dynamic communication with systemic metabolic and immunological processes through multiple pathways collectively termed the neuro–immune–endocrine axis (Dantzer et al., 2008; Engelhardt et al., 2017). This integrated network facilitates bidirectional signaling between the CNS and peripheral tissues, including adipose depots, the liver, pancreas, and the immune system, enabling dynamic regulation of energy balance, inflammation, and stress responses (Pavlov and Tracey, 2017). Disruption of this finely tuned communication network in states of metabolic excess contributes to neuroinflammation, synaptic dysfunction, and cognitive impairment (Le Thuc and García-Cáceres, 2024; Miller and Spencer, 2014).

Metabolic-associated steatotic liver disease (MASLD), formerly known as non-alcoholic fatty liver disease (NAFLD), represents a particularly important mediator linking peripheral metabolic dysfunction to CNS pathology. MASLD has been associated with reduced brain volume, altered white-matter integrity, and increased dementia risk (Weinstein et al., 2018). Affecting approximately 30% of adults worldwide, MASLD encompasses a spectrum of hepatic pathology ranging from simple steatosis to metabolic dysfunction-associated steatohepatitis (MASH) with varying degrees of fibrosis, potentially progressing to cirrhosis and hepatocellular carcinoma (Mikkelsen et al., 2025; Younossi et al., 2016). Recent observational and imaging data indicate that MASLD is associated with neuroinflammatory signaling, functional brain alterations, and higher rates of cognitive impairment, although the extent to which these relationships are independent of shared cardiometabolic and vascular risk factors remains under active investigation (Mikkelsen et al., 2025). Evidence from animal models supports a causal hepato-neural axis whereby liver-specific pathology can induce hippocampal dysfunction and behavioral changes, but human data are still largely associative (Huang et al., 2025). In addition, neuroimaging studies show that fibrosis severity in MASLD correlates with white matter lesions, highlighting increased cerebrovascular vulnerability (Petta et al., 2016).

This review comprehensively examines the molecular and cellular mechanisms underlying the detrimental impact of obesity, MASLD, and broader metabolic dysregulation on brain health. We detail how alterations in adipokine profiles, systemic inflammatory mediators, and endocrine signaling pathways collectively disrupt neuro–immune–endocrine homeostasis, promoting neuroinflammation, neuronal dysfunction, and cognitive deterioration. Furthermore, we explore the emerging concept of “metabolic-associated cognitive decline” as a potentially preventable or reversible neurological consequence of metabolic disorders (Beilharz et al., 2015). By elucidating these complex pathways, we highlight therapeutic opportunities to mitigate neurological complications in metabolically compromised individuals Understanding the intricate relationship between metabolic health and neurological function represents a crucial frontier in addressing the neurological sequelae of the global obesity epidemic.

## 3 The neuro–immune–endocrine axis: anatomical and functional organization

### 3.1 Anatomical foundations of neuro–immune–endocrine integration

The neuro–immune–endocrine axis represents a highly integrated network that coordinates physiological responses to environmental challenges and maintains systemic homeostasis (Besedovsky and Rey, 2007; Bower and Kuhlman, 2023). This axis is anchored by three primary systems: the central nervous system (CNS), the immune system, and the endocrine system interfaces. These systems converge within specialized “neuroimmune hubs,” where metabolic, hormonal, and inflammatory signals are processed to shape whole-body physiology (Bower and Kuhlman, 2023; Chrousos, 2009; Sternberg, 2006). The hypothalamus serves as the central integrator of this axis, receiving and processing signals from peripheral tissues and orchestrating appropriate neural, immune, and endocrine responses (Besedovsky and Rey, 2007; Saper and Lowell, 2014). Key hypothalamic nuclei, including the arcuate, paraventricular, and ventromedial nuclei, express receptors for hormones, cytokines, and other signaling molecules that convey information about peripheral metabolic and immune status (Sternberg, 2006; Saper and Lowell, 2014). These nuclei project to other brain regions involved in cognition, mood regulation, autonomic control, and energy balance, including the hippocampus, amygdala, prefrontal cortex, and brainstem nuclei (Saper and Lowell, 2014; Myers and Olson, 2012). The circumventricular organs (CVOs), including the median eminence, organum vasculosum of the lamina terminalis, subfornical organ, and area postrema, represent crucial sites of neuro–immune–endocrine interaction (Black et al., 2017). These specialized brain regions lack a complete blood-brain barrier (BBB), allowing direct sampling of circulating hormones, cytokines, nutrients, and immune mediators by endothelial, glial, and neuronal cells within the CVOs (Saper and Lowell, 2014; Langlet et al., 2013). This anatomical arrangement facilitates rapid neural detection of peripheral metabolic perturbations and provides a substrate for immune-to-brain signaling (Balland et al., 2014). The autonomic nervous system (ANS), comprising sympathetic and parasympathetic branches, forms additional bidirectional connections between the CNS and peripheral tissues, including adipose tissue, liver, pancreas, gut, and lymphoid organs (Rosas-Ballina and Tracey, 2009), providing rapid CNS modulation of metabolism and immunity, while afferent pathways convey information about peripheral inflammation, nutrient status, and organ function back to the CNS (Pavlov and Tracey, 2012; Brestoff and Artis, 2015). Through these pathways, the ANS participates in reflexive regulation of immunity, such as the inflammatory reflex, and the fine-tuning of metabolic homeostasis.

### 3.2 Molecular mediators of neuro–immune–endocrine communication

Communication across the neuro–immune–endocrine axis is mediated by a diverse array of molecular signals that are recognized by multiple cell types across these systems. These mediators include:

*Cytokines and chemokines:* initially characterized as immune signaling molecules, cytokines such as interleukin-1β (IL-1β), interleukin-6 (IL-6), tumor necrosis factor-α (TNF-α), and chemokines like CCL2 (MCP-1) are now recognized to profoundly influence neural endocrine function (Dinarello, 2009; Schett et al., 2013). These factors can signal across the BBB through active transport mechanisms, act on CVOs, or induce endothelial production of second messengers that relay immune signals to the brain (Banks, 2015).

*Adipokines:* adipose tissue-derived hormones, including leptin, adiponectin, resistin, and visfatin, exert pleiotropic effects on metabolic, immune, and neural functions (Kwon and Pessin, 2013). Leptin, in particular, acts as a crucial regulator of energy homeostasis by signaling through hypothalamic receptors, while also modulating immune cell function and broader neural processes (Friedman, 2011; La Cava and Matarese, 2004). Dysregulated adipokine signaling bridges obesity, low-grade inflammation, and neuroendocrine remodeling (Friedman, 2011; La Cava and Matarese, 2004).

*Classical hormones:* glucocorticoids, thyroid hormones, growth hormone, and sex steroids regulate multiple aspects of metabolism, immune function, and neural development/ plasticity (McEwen, 1992; McEwen and Akil, 2020). These hormones typically signal through nuclear receptors that modulate gene expression, although non-genomic signaling mechanisms also contribute to their effects (Kino and Chrousos, 2004; Pariante and Lightman, 2008). HPA axis dynamics (CRH–ACTH–cortisol) exemplify stress-responsive endocrine control of neuroimmune tone effects (Pariante and Lightman, 2008).

*Neurotransmitters and neuropeptides*: catecholamines (epinephrine, norepinephrine), acetylcholine, serotonin, and neuropeptides (e.g., neuropeptide Y, orexin, melanocortins) regulate both neural activity and immune cell function (Dantzer, 2018). Immune cells express receptors for these neural signaling molecules and can synthesize select neurotransmitters, creating bidirectional crosstalk (Kenney and Ganta, 2014; Glaser and Kiecolt-Glaser, 2005).

*Damage-associated molecular patterns (DAMPs):* molecules released from damaged or stressed cells, including high mobility group box 1 (HMGB1), heat shock proteins, and extracellular ATP, activate pattern recognition receptors on immune and neural cells (particularly microglia and astrocytes), triggering inflammatory responses (Qin et al., 2006; Vénéreau et al., 2015). Extracellular ATP activates P2X7 receptors on microglia to drive inflammasome signaling, whereas HMGB1 signals through innate immune pattern-recognition receptors, including Toll-like receptors and the receptor for advanced glycation end products (RAGE), triggering neuroinflammation after tissue injury (Bianchi et al., 2017; Tewari et al., 2024).

*Metabolic intermediates:* nutrients and metabolites, including glucose, free fatty acids, ketone bodies, and lactate, signal cellular nutrient status via mTOR/AMPK and metabolite-sensing receptors to immune and neural cells (O'Neill et al., 2016; Fang et al., 2024; García-Rodríguez and Giménez-Cassina, 2021; Llibre et al., 2025). Lactate acts as both fuel and paracrine immunomodulator with context-dependent pro- and anti-inflammatory effects (O'Neill et al., 2016; Fang et al., 2024; García-Rodríguez and Giménez-Cassina, 2021; Llibre et al., 2025).

*Microbiota-derived metabolites:* short-chain fatty acids (SCFAs), tryptophan metabolites, and secondary bile acid derivatives produced by the intestinal microbiota influence neuro–immune–endocrine signaling by regulating immune cell differentiation, microglial activation, and neuronal signaling pathways, thereby contributing to gut–brain communication and systemic metabolic homeostasis (Koh et al., 2016; Agus et al., 2018). SCFAs such as butyrate and propionate influence microglial maturation and inflammatory tone, while tryptophan metabolites regulate intestinal and CNS immune responses (Rothhammer et al., 2016; Erny et al., 2015).

*Bile acids:* beyond their classical role in lipid digestion, bile acids function as endocrine/ immunomodulatory cues through FXR- and TGR5-dependent signaling, linking liver, gut, and brain, with system-wide metabolic and inflammatory effects, modulating metabolic, immune, and neural pathways (Fiorucci et al., 2018; Wahlström et al., 2016).

*Lipid mediators:* bioactive lipid mediators, including prostaglandins and leukotrienes, initiate and amplify inflammation, whereas specialized pro-resolving mediators (resolvins, protectins, lipoxins) actively terminate it, shaping glial activation, neuronal excitability, and cytokine production, linking metabolic stress to CNS immune responses (Serhan, 2014; Dennis and Norris, 2015).

*Growth factors:* growth factors such as brain-derived neurotrophic factor (BDNF), nerve growth factor (NGF), and insulin-like growth factor-1 (IGF-1) regulate neuronal survival, synaptic plasticity, and immune cell function, providing a molecular bridge between neural activity and immune regulation. These factors respond dynamically to metabolic and inflammatory cues, enabling coordinated neuro–immune communication (Bathina and Das, 2015; Carro et al., 2000).

*Extracellular vesicles:* exosomes and other microvesicles released from immune, endocrine, and neural cells transport proteins, lipids, and regulatory RNAs between tissues and can cross the BBB serving as an important mechanism of long-distance neuro–immune–endocrine communication and systemic responses to focal CNS injury (Yáñez-Mó et al., 2015; Dickens et al., 2017).

Together, these molecular mediators form an integrated biochemical network that enables rapid and adaptive communication across the neuro–immune–endocrine axis, providing the mechanistic foundation through which metabolic perturbations influence CNS function and systemic physiology.

### 3.3 Cellular components of the *neuro*–*immune*–*endocrine* axis

Multiple specialized cell types contribute to neuro–immune–endocrine integration and respond to perturbations in metabolic homeostasis. Together, these cells form the functional substrate through which peripheral metabolic and inflammatory cues influence CNS activity, and through which the brain regulates systemic physiology.

*Microglia*: as the resident macrophages of the CNS, microglia continuously survey the neural microenvironment and rapidly respond to inflammatory signals, metabolic alterations, and neural injury (Prinz and Priller, 2014). Through distinct activation states, microglia exert significant influences on neuronal function, synaptic plasticity, and cognitive processes (Salter and Stevens, 2017; Wolf et al., 2017; Borst et al., 2021). Microglia express receptors for glucocorticoids, leptin, insulin, and multiple cytokines, positioning them as key cellular nodes in neuro–immune–endocrine integration and enabling rapid immune modulation in response to metabolic cues (Bower and Kuhlman, 2023; Chrousos, 2009; Sternberg, 2006; Borst et al., 2021). Microglia also undergo metabolic reprogramming during overnutrition, shifting toward glycolytic, pro-inflammatory phenotypes that amplify hypothalamic inflammation and disrupt energy-balance circuits (Salter and Stevens, 2017; Wolf et al., 2017; Borst et al., 2021).

*Astrocytes:* these multifunctional glial cells play essential roles in BBB maintenance, regulating neurotransmitter uptake and recycling, providing metabolic substrates to neurons, and releasing trophic factors for neurons (Allen and Eroglu, 2017). Astrocytes express receptors for numerous cytokines, adipokines, and metabolic signals, allowing them to detect systemic metabolic disturbances and convert immune and endocrine cues into changes in neuronal and synaptic function (Sofroniew and Vinters, 2010; Liddelow et al., 2017). Through their roles in glucose and lactate regulation, astrocytes also act as key metabolic intermediaries during inflammation or nutrient excess, supporting neuronal energy demands while shaping local immune responses (García-Rodríguez and Giménez-Cassina, 2021; Llibre et al., 2025; Allen and Eroglu, 2017).

*Tanycytes:* specialized ependymal-like glial cells lining the third ventricle, tanycytes detect circulating metabolic signals and convey this information to hypothalamic neurons involved in energy homeostasis (Bolborea and Dale, 2013). By expressing transporters and receptors for circulating factors like glucose, leptin, and insulin, tanycytes function as metabolic sensors and gatekeepers that regulate the entry of peripheral signals into the metabolic hypothalamus (Langlet et al., 2013; Langlet, 2014). Their strategic location at the interface between the cerebrospinal fluid and hypothalamic parenchyma enables controlled transport of metabolic and hormonal signals into the brain, providing a structural pathway linking peripheral metabolic and immune signals to central hypothalamic responses (Balland et al., 2014).

*Endothelial cells:* cerebrovascular endothelial cells form the structural basis of the BBB and express a wide range of transporters and receptors for metabolic and inflammatory mediators (Zlokovic, 2008; Daneman and Prat, 2015). Beyond their barrier function, endothelial cells actively participate in neuro–immune–endocrine communication by conveying peripheral signals to the CNS through the production of second messengers and regulated transport processes (Daneman and Prat, 2015; Abbott et al., 2006). Endothelial dysfunction during metabolic disease can impair BBB integrity and amplify neuroinflammatory signaling, thereby altering CNS responses to peripheral metabolic cues (Zlokovic, 2008; Daneman and Prat, 2015; Abbott et al., 2006).

*Perivascular macrophages and mast cells:* these CNS-associated immune cells reside in perivascular and meningeal spaces, where they respond to peripheral immune and metabolic signals. Perivascular *macrophages* contribute to neurovascular dysfunction and cognitive impairment by amplifying inflammatory signaling and promoting oxidative stress (Faraco et al., 2016; Theoharides, 2017; Skaper et al., 2014). Whereas meningeal and perivascular *mast* cells modulate BBB permeability and neuroinflammatory responses through the release of histamine, cytokines (e.g., TNF-α, IL-1β), proteases (e.g., tryptase, chymase), and other vasoactive mediators (Faraco et al., 2016; Theoharides, 2017; Skaper et al., 2014). Together, these cells act as key intermediaries between the circulation and the brain, shaping vascular, immune, and neuronal responses during metabolic stress.

*Hypothalamic neurons:* specialized neuronal populations in the arcuate nucleus in the hypothalamus, including proopiomelanocortin (POMC) and agouti-related peptide (AgRP) neurons, integrate metabolic, immune, and endocrine signals to regulate energy homeostasis and other physiological processes (Timper and Brüning, 2017; Waterson and Horvath, 2015). By sensing circulating nutrients (e.g., glucose, fatty acids), hormones (e.g., leptin, insulin, GLP-1), and inflammatory cytokines, they coordinate feeding behavior, energy expenditure, glucose regulation, and autonomic output, while also influencing cardiovascular function and systemic inflammatory responses (Timper and Brüning, 2017; Waterson and Horvath, 2015).

*Adipocytes*: adipocytes function not only as lipid-storing cells but also as endocrine and immunometabolic effectors that secrete adipokines, cytokines, and lipid mediators, enabling adipose tissue to communicate metabolic status to the brain and immune system (Ouchi et al., 2011; Rosen and Spiegelman, 2014; Hotamisligil, 2017). In obesity, adipocytes hypertrophy and metabolic stress promote increased release of pro-inflammatory cytokines (e.g., TNF, IL-6) and altered adipokine profiles, thereby driving systemic inflammation and insulin resistance (Ouchi et al., 2011; Hotamisligil, 2017). These inflammatory and hormonal signals influence hypothalamic circuits involved in energy balance and contribute to neuroinflammatory processes that impair metabolic regulation (Thaler et al., 2012).

*Neuroendocrine cells*: neuroendocrine components of the hypothalamic–pituitary–adrenal axis provide a major hormonal interface linking stress, metabolism, and immune regulation (Herman et al., 2016). Glucocorticoids, and pituitary-derived hormones act on hypothalamic and brainstem circuits to coordinate energy homeostasis, stress responses, and inflammatory tone, while leptin signaling further interfaces with hypothalamic homeostatic circuits (Herman et al., 2016; Myers et al., 2008).

*Enteroendocrine cells and GLP-1 signaling components*: enteroendocrine cells in the gastrointestinal epithelium sense luminal nutrients and release hormones such as Glucagon-like peptide-1 (GLP-1), peptide YY, ghrelin, and cholecystokinin, which influence hypothalamic circuits and immune pathways, and thereby link gut nutrient sensing to central metabolic regulation (Gribble and Reimann, 2016; Clemmensen et al., 2017). Among these pathways, GLP-1 signaling is particularly important. GLP-1 is produced by intestinal enteroendocrine L cells and preproglucagon-expressing neurons in the nucleus tractus solitarius, and acts on GLP-1-responsive neurons distributed across hypothalamic and brainstem nuclei to regulate satiation, glucose homeostasis, energy balance, and autonomic function (Gribble and Reimann, 2016; Clemmensen et al., 2017; Pais et al., 2016; Müller et al., 2019). In addition to metabolic effects, GLP-1 signaling exerts immunomodulatory actions by influencing inflammatory pathways and immune cell activity, thereby linking gut-derived signals with central and systemic control of metabolic and inflammatory homeostasis (Brestoff and Artis, 2015; Saltiel and Olefsky, 2017).

*Peripheral immune cells*: circulating immune populations, including T cells (CD4+, CD8+, and regulatory T cells), B cells, natural killer cells, and neutrophils, contribute to neuro–immune–endocrine crosstalk by producing cytokines and interacting with neural and endocrine pathways (Saltiel and Olefsky, 2017; McLaughlin et al., 2017; Wensveen et al., 2015; Jacks and Lumeng, 2024). These cells respond dynamically to metabolic and hormonal signals and, in turn, influence systemic inflammation, insulin sensitivity, and tissue homeostasis, linking peripheral immune activity to central metabolic regulation and disease processes (McLaughlin et al., 2017). In obesity and metabolic dysfunction, these cells adopt pro-inflammatory phenotypes that amplify systemic inflammation, impair insulin sensitivity, and modulate hypothalamic and autonomic pathways involved in energy balance and metabolic control (Wensveen et al., 2015; Jacks and Lumeng, 2024). This anatomical, cellular, and functional organization of the neuro–immune–endocrine axis provides the foundation for understanding how metabolic perturbations associated with obesity and MASLD impact neuroinflammation and cognitive function, as will be elaborated in subsequent sections.

## 4 Metabolic dysfunction and systemic inflammation: the peripheral inflammatory milieu

### 4.1 Adipose tissue dysfunction in obesity: beyond fat storage

Adipose tissue is now recognized as an active endocrine organ that releases adipokines involved in metabolic homeostasis and obesity-associated inflammation (Kershaw and Flier, 2004; Nakamura et al., 2014). In obesity, adipose tissue undergoes significant structural and functional remodeling that promotes metabolic dysfunction and inflammatory activation (Boutens and Stienstra, 2016). These alterations are thought to contribute to pathways linking obesity to neuroinflammation and cognitive impairment (Kiliaan et al., 2014). Obesity is also associated with adipocyte dysfunction and altered adipokine secretion, both of which promote inflammation and metabolic disease (Ouchi et al., 2011). This hypertrophic expansion is accompanied by reduced vascular density relative to tissue mass, leading to hypoxic regions within adipose depots (Sun et al., 2013). In this setting, activation of hypoxia-inducible factor-1α (HIF-1α) promotes pro-inflammatory gene expression and worsens adipocyte stress and dysfunction. In turn, this can contribute to cell death and amplify local inflammatory responses (Lee et al., 2014; Odegaard and Chawla, 2013). A hallmark of adipose tissue dysfunction in obesity is a marked shift in resident immune cell populations (Lumeng and Saltiel, 2011). In lean adipose tissue, anti-inflammatory immune cells, including alternatively activated (M2-like) macrophages and regulatory T cells (Tregs), contribute to maintaining tissue homeostasis (Olefsky and Glass, 2010). In contrast, obese adipose tissue is characterized by increased accumulation of pro-inflammatory immune cells, including inflammatory macrophages, neutrophils, CD8^+^ T cells, and B cells, along with a reduction in Tregs and eosinophils (Cildir et al., 2013; Winer et al., 2011). Part of this immune cell recruitment is driven by adipocyte-derived chemokines, especially CCL2 (MCP-1), which attract circulating monocytes that later adopt an inflammatory macrophage phenotype (Kanda et al., 2006). Adipose tissue macrophages in obesity form crown-like structures surrounding dead or dying adipocytes and secrete pro-inflammatory cytokines, including TNF-α, IL-1β, and IL-6 (Cinti et al., 2005; Lumeng et al., 2007). These macrophages also generate reactive oxygen and nitrogen species that contribute to local tissue damage (Furukawa et al., 2004). This inflammatory milieu impairs adipocyte insulin sensitivity and enhances lipolysis. The resulting increase in free fatty acid release further activates inflammatory pathways through toll-like receptors (TLRs) and the NLRP3 inflammasome, reinforcing a cycle of inflammation and metabolic dysfunction (Vandanmagsar et al., 2011; Shi et al., 2006). Among adipose-derived factors, TNF-α, IL-6, IL-1β and chemokines such as CCL2 are particularly relevant to brain function because they can signal across the BBB or via circumventricular organs, where they promote microglial activation, astrocytic reactivity, and altered neuroendocrine signaling. Taken together, these findings show that adipose tissue in obesity is far more than an expanded energy store. It becomes an active source of inflammatory and metabolic signals with effects that extend to both peripheral tissues and the brain.

### 4.2 Adipokine dysregulation: altered endocrine signaling in obesity

A major consequence of adipose dysfunction in obesity is altered adipokine signaling. These bioactive mediators regulate systemic metabolism, immune function, and neuroendocrine pathways (Lehr et al., 2012). In obesity, adipokine production and secretion become dysregulated, contributing to both peripheral and central inflammation (Coelho et al., 2013; Fasshauer and Blüher, 2015; Myers et al., 2010). Leptin, the product of the ob gene, is a key regulator of energy homeostasis that signals nutritional status to the central nervous system (Myers et al., 2010). Circulating leptin levels correlate with adipose tissue mass, and obesity is characterized by hyperleptinemia accompanied by central leptin resistance (Jung and Kim, 2013). Leptin also has important immunomodulatory effects, enhancing T-cell proliferation, promoting T-helper 1 (Th1) responses, and stimulating macrophage production of pro-inflammatory cytokines (Lord et al., 1998; Matarese et al., 2010). Within the central nervous system, leptin activates JAK-STAT and PI3K-Akt signaling pathways in neurons and glial cells, influencing neurogenesis, synaptic plasticity, and inflammatory responses (Myers et al., 2008). Hyperleptinemia in obesity may contribute to neuroinflammation despite reduced central metabolic responsiveness to leptin (Paz-Filho et al., 2010; Signore et al., 2008). In contrast, adiponectin levels inversely correlate with adiposity, resulting in hypoadiponectinemia in obesity (Kadowaki et al., 2006). Adiponectin exerts anti-inflammatory effects, including suppression of macrophage TNF-α production, inhibition of NF-κB activation, and promotion of anti-inflammatory cytokines such as IL-10 and IL-1 receptor antagonist (Ohashi et al., 2010; Yokota et al., 2000). Adiponectin promotes insulin sensitivity and fatty acid oxidation through activation of AMP-activated protein kinase (AMPK) (Yamauchi et al., 2002). Within the central nervous system, adiponectin exerts neuroprotective effects, including suppression of microglial activation and reduction of oxidative stress (Chen et al., 2009; Ng and Chan, 2017). Reduced adiponectin levels in obesity may therefore favor a more pro-inflammatory environment in the brain (Thundyil et al., 2012). Other adipokines are also altered in obesity. These include resistin, which promotes insulin resistance and inflammation through NF-κB-dependent pathways (Steppan et al., 2001), visfatin, which has both metabolic and pro-inflammatory properties (Chang et al., 2011), and chemerin, which acts as both an adipokine and a chemokine and helps regulate immune cell recruitment to sites of inflammation (Bozaoglu et al., 2007; Rourke et al., 2013). Overall, the adipokine profile of obesity shifts away from metabolic homeostasis and toward inflammation, providing another route by which peripheral metabolic dysfunction can affect the brain (Tilg and Moschen, 2006).

### 4.3 Hepatic inflammation in MASLD: the liver–brain inflammatory axis

MASLD is a common hepatic manifestation of obesity-related metabolic dysfunction and encompasses a spectrum of pathology ranging from steatosis to steatohepatitis, fibrosis, cirrhosis, and hepatocellular carcinoma (Friedman et al., 2018; International Consensus Panel, 2020). Across the spectrum from simple steatosis to MASH, advanced fibrosis, and cirrhosis, distinct mechanisms link liver pathology to brain dysfunction, including systemic inflammation, altered ammonia and amino acid handling, bile acid signaling, and gut-derived factors. Advanced fibrosis and cirrhosis additionally increase risk of overt hepatic encephalopathy and cerebrovascular complications, whereas earlier MASLD stages may exert subtler effects on cognition through low-grade inflammation and vascular risk. MASLD is strongly associated with obesity, insulin resistance, and type 2 diabetes, and affects approximately 30% of the global adult population (Younossi et al., 2016). Emerging evidence suggests that MASLD may contribute to neuroinflammation and cognitive dysfunction through hepatic release of inflammatory mediators, metabolic alterations, and disruption of gut–liver–brain signaling pathways (Tilg and Moschen, 2010; Seo et al., 2016). The pathogenesis of MASLD begins with excessive hepatic lipid accumulation, particularly triglycerides, driven by increased free fatty acid flux from adipose tissue, enhanced *de novo* lipogenesis, reduced fatty acid oxidation, and impaired very low-density lipoprotein (VLDL) secretion (Postic and Girard, 2008; Donnelly et al., 2005). This lipid accumulation, particularly of saturated fatty acids and free cholesterol, induces hepatocellular stress responses, including endoplasmic reticulum (ER) stress and oxidative stress associated with mitochondrial dysfunction and increased reactive oxygen species (ROS) production (Lebeaupin et al., 2018). These cellular stresses activate inflammatory signaling cascades, including NF-κB and JNK pathways, leading to increased production of pro-inflammatory cytokines and chemokines by hepatocytes (Cai et al., 2005; Sabio et al., 2008). Hepatic inflammation in MASLD is further amplified by activation of Kupffer cells, the resident macrophages of the liver (Dixon et al., 2013). Kupffer cells respond to lipotoxic injury by producing inflammatory cytokines (TNF-α, IL-1β, IL-6) and chemokines (e.g., CCL2), which promote recruitment of additional inflammatory cells, including circulating monocytes that differentiate into macrophages, as well as neutrophils and lymphocyte populations (Huang et al., 2010; Tacke and Zimmermann, 2014). This inflammatory cascade contributes to the development of steatohepatitis and progression to fibrosis through activation of hepatic stellate cells (Friedman, 2008). The inflamed liver in MASLD releases inflammatory mediators into the systemic circulation that may impact central nervous system function (Huang et al., 2025). Elevated hepatic production and circulating levels of TNF-α, IL-6, IL-1β, and various chemokines have been documented in MASLD, with levels correlating with disease severity (Koyama and Brenner, 2017; Wree et al., 2013). These cytokines may influence the brain through several routes, including transport across the blood–brain barrier (BBB), activation of vagal afferents, endothelial signaling, and access through circumventricular organs that lack a fully intact BBB (Banks and Erickson, 2010; D'Mello and Swain, 2014). Additionally, MASLD is associated with altered metabolism of neurotoxic compounds (Aldridge et al., 2015). The liver normally detoxifies ammonia through the urea cycle, but hepatic dysfunction can lead to hyperammonemia, which can cross the BBB and disrupt neurotransmission (Felipo and Butterworth, 2002). Similarly, impaired hepatic metabolism of aromatic amino acids alters their ratio to branched-chain amino acids, potentially affecting neurotransmitter synthesis (Dejong et al., 2007). MASLD is also associated with elevated circulating bile acids, which can cross the blood–brain barrier and act as signaling molecules in the central nervous system (McMillin and DeMorrow, 2016; MahmoudianDehkordi et al., 2019). Recent evidence highlights the importance of the gut–liver–brain axis in MASLD pathophysiology (Tripathi et al., 2018). Obesity and MASLD are associated with intestinal dysbiosis, increased gut permeability, and translocation of bacterial products (e.g., lipopolysaccharide, peptidoglycans) into the portal circulation (Cani et al., 2007; Brun et al., 2007). These microbial products activate hepatic inflammatory signaling through pattern recognition receptors and contribute to MASLD progression (Miura and Ohnishi, 2014). Furthermore, microbial metabolites altered in MASLD, including short-chain fatty acids and trimethylamine N-oxide (TMAO), may influence neuroinflammation and cognitive function (Zhu et al., 2016; Hoyles et al., 2018). Several longitudinal studies suggest that MASLD and liver fibrosis are associated with changes in cognitive performance and dementia risk, but findings are heterogeneous and sometimes divergent across stages and populations, necessitating the need for careful staging and adjustment for vascular and metabolic confounders. In this context, MASLD is more than a hepatic manifestation of metabolic disease; it represents a source of inflammatory, metabolic, and gut-derived signals that may influence brain function. However, longitudinal and interventional studies are still needed to clarify when MASLD acts as an independent driver vs. a marker of broader metabolic risk.

### 4.4 Insulin resistance and hyperglycemia: metabolic drivers of inflammation

Insulin resistance is a core feature of obesity, metabolic syndrome, and type 2 diabetes, and it is closely linked to the systemic inflammatory environment that may affect neurological function (Shoelson et al., 2006; Gregor and Hotamisligil, 2011). In insulin-resistant states, impaired cellular responses to insulin disrupt glucose homeostasis and lipid metabolism, creating metabolic conditions that activate inflammatory signaling pathways across multiple tissues (Olefsky and Glass, 2010). At the molecular level, insulin resistance arises through several interconnected mechanisms (Samuel and Shulman, 2016). Pro-inflammatory cytokines, particularly TNF-α and IL-1β, impair insulin signaling by activating JNK and IKK-β, which phosphorylate insulin receptor substrate-1 (IRS-1) at inhibitory serine residues (Aguirre et al., 2000). This modification interferes with the normal tyrosine phosphorylation required for downstream insulin signaling (Gao et al., 2002; Copps and White, 2012). Additionally, suppressor of cytokine signaling-3 (SOCS-3), induced by inflammatory cytokines, interferes with insulin receptor signaling and promotes proteasomal degradation of insulin receptor substrate (IRS) proteins (Ueki et al., 2004). Free fatty acids are also elevated in obesity as lipolysis increases in insulin-resistant adipose tissue. These fatty acids activate inflammatory signaling through TLR4 and promote accumulation of lipid intermediates such as diacylglycerols and ceramides, which further impair insulin signaling (Holland et al., 2011; Samuel et al., 2010). Hyperglycemia, resulting from insulin resistance and pancreatic β-cell dysfunction, further exacerbates inflammatory activation through multiple mechanisms (Giacco and Brownlee, 2010). Chronic hyperglycemia promotes formation of advanced glycation end-products (AGEs), which activate the receptor for AGEs (RAGE) and trigger NF-κB-mediated inflammatory signaling (Vlassara and Striker, 2011; Goldin et al., 2006). Hyperglycemia also increases reactive oxygen species (ROS) production through enhanced mitochondrial electron transport chain activity and activation of the polyol pathway (Rolo and Palmeira, 2006). This promotes oxidative stress and further inflammatory signaling. Furthermore, hyperglycemia increases flux through the hexosamine biosynthetic pathway, resulting in O-linked N-acetylglucosamine (O-GlcNAc) modification of transcription factors that regulate inflammatory gene expression (Hart et al., 2007; Yang et al., 2008). Within the central nervous system, insulin resistance and hyperglycemia are associated with neuroinflammation and cognitive dysfunction (Felice and Ferreira, 2014). Insulin receptors and insulin-sensitive glucose transporters are widely expressed in the brain, particularly in regions involved in cognitive function such as the hippocampus and cerebral cortex (Zhao and Alkon, 2001; Kleinridders et al., 2014). Neuronal insulin resistance impairs synaptic plasticity, neurotransmitter release, and dendritic spine formation (Chiu et al., 2008). Hyperglycemia also induces oxidative stress and inflammatory activation in cerebral endothelial cells, pericytes, and astrocytes. These changes can compromise blood–brain barrier (BBB) integrity and facilitate the entry of peripheral inflammatory mediators into the central nervous system (Brownlee, 2001; Stranahan et al., 2016). Additionally, hyperglycemia enhances microglial reactivity to inflammatory stimuli, thereby potentiating neuroinflammatory responses (Hwang et al., 2014). Taken together, systemic inflammation, adipokine dysregulation, hepatic inflammation, and insulin resistance with hyperglycemia shape a peripheral inflammatory environment that may influence central nervous system function. The next section examines how these peripheral signals may drive neuroinflammation and cognitive impairment through disruption of neuro–immune–endocrine pathways.

## 5 Neuroinflammation in metabolic disease: mechanisms and consequences

### 5.1 Hypothalamic inflammation: the sentinel response to metabolic stress

The hypothalamus, particularly its mediobasal region (encompassing the arcuate nucleus and median eminence), serves a primary CNS site of inflammation in response to obesity and metabolic dysfunction (Thaler et al., 2012; Valdearcos et al., 2014). Its proximity to the median eminence—a circumventricular organ lacking a complete blood-brain barrier (BBB)—and its role in detecting circulating metabolic signals heighten vulnerability to peripheral inflammatory mediators (Thaler et al., 2012; Yi and Tschöp, 2012). High-fat diet exposure triggers rapid hypothalamic inflammation, often preceding weight gain and peripheral inflammation, positioning it as an early driver of obesity (Posey et al., 2009; Dalvi et al., 2017).

**Figure 1:**
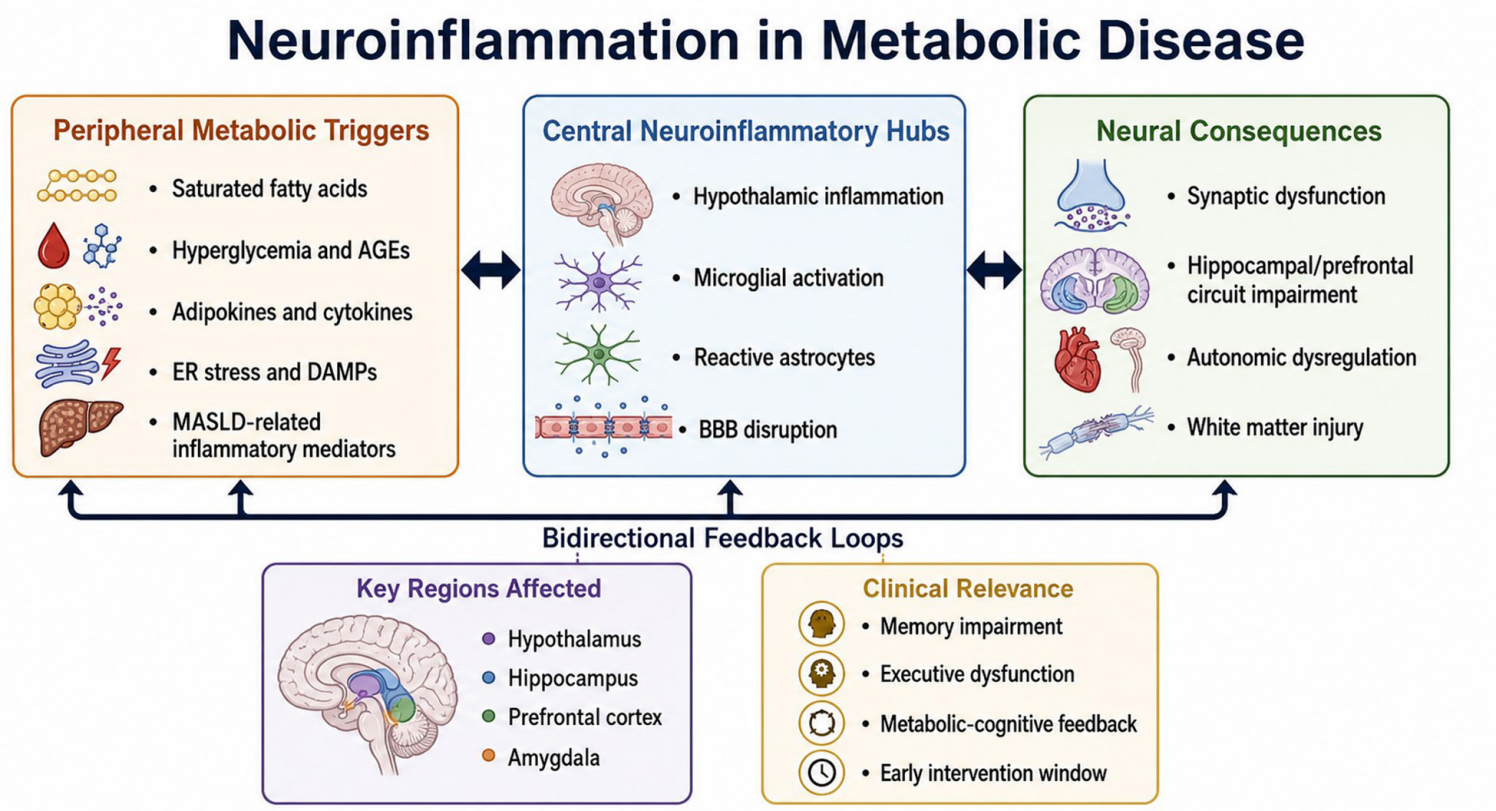
Neuroinflammation in metabolic disease. Peripheral metabolic triggers (including adipokines, cytokines, MASLD-related mediators, hyperglycemia/AGEs, and ER stress/DAMPs) interact bidirectionally with central neuroinflammatory hubs (hypothalamic inflammation, microglial activation, reactive astrocytes, and blood–brain barrier disruption), leading to neural consequences such as synaptic dysfunction, hippocampal–prefrontal circuit impairment, autonomic dysregulation, and white matter injury. Key regions affected include the hypothalamus, hippocampus, prefrontal cortex, and amygdala, with clinical manifestations encompassing memory impairment, executive dysfunction, metabolic–cognitive feedback loops, and an early intervention window.

Multiple metabolic triggers initiate this process (Figure 1). Mechanistically, hypothalamic inflammation is initiated by multiple triggers associated with metabolic excess (Zhang et al., 2008). Saturated fatty acids, particularly palmitate, activate TLR4 signaling in microglia and astrocytes, leading to NF-κB-mediated production of pro-inflammatory cytokines (Valdearcos et al., 2014; Milanski et al., 2009). Chronic hyperleptinemia engages Janus kinase-signal transducer and activator of transcription 3 (JAK-STAT3) pathways, shifting from homeostatic to pro-inflammatory roles (Hübschle et al., 2001; Liu et al., 2021). Advanced glycation end-products (AGEs)-formed under hyperglycemic conditions- activate RAGE and stimulate downstream inflammatory signaling (Yan et al., 2008). Additionally, ER stress induced by nutrient excess activates the unfolded protein response in hypothalamic neurons, promoting inflammatory signaling and metabolic dysfunction (Zhang et al., 2008; Won et al., 2009; Williams et al., 2014). The inflammatory response in the hypothalamus is characterized by reactive gliosis, with both microglia and astrocytes exhibiting activated phenotypes (Valdearcos et al., 2014; Buckman et al., 2013). Microgliosis, in particular, represents a hallmark of hypothalamic inflammation in obesity, with microglia displaying enlarged soma, shortened processes, and enhanced production of inflammatory cytokines (Valdearcos et al., 2017; Gao et al., 2014). Intriguingly, recent evidence suggests that hypothalamic microgliosis may exhibit both detrimental and beneficial effects on metabolic regulation (Valdearcos et al., 2017). While prolonged microglial activation contributes to hypothalamic neuronal dysfunction and resistance to anorexigenic signals, acute microglial activation may also dynamically influence metabolic circuits (Jha et al., 2015; García-Cáceres et al., 2012; Reis et al., 2015). Specifically, activated microglia modulate POMC neuron function through TNF α-dependent increases in mitochondrial activity and neuronal excitability, highlighting the role of microglia in regulating anorexigenic signaling (Valdearcos et al., 2017; Reis et al., 2015; Yi et al., 2017). Astrocytes in the obese hypothalamus also adopt reactive phenotypes, characterized by hypertrophy, increased glial fibrillary acidic protein (GFAP) expression, and production of inflammatory cytokines and chemokines (Horvath et al., 2010; Douglass et al., 2017). Reactive astrocytes contribute to BBB disruption through altered expression of tight junction proteins, potentially facilitating further influx of peripheral inflammatory mediators into the CNS (Douglass et al., 2017; Argente-Arizón et al., 2015). Hypothalamic inflammation significantly impacts neuronal populations involved in energy homeostasis regulation (Thaler et al., 2012; Baquero et al., 2014). POMC neurons, which produce the anorexigenic neuropeptide α-melanocyte-stimulating hormone (α-MSH), exhibit impaired leptin and insulin sensitivity, reduced neurite outgrowth, and altered synaptic organization under inflammatory conditions (Thaler et al., 2012; Zeltser et al., 2012). Conversely, orexigenic agouti-related peptide (AgRP) neurons may initially exhibit enhanced activity in response to inflammatory stimuli, promoting hyperphagia (Reis et al., 2015; García-Cáceres et al., 2014). Prolonged inflammation leads to disruption of hypothalamic circuitry regulating energy balance, contributing to obesity persistence despite elevated levels of anorexigenic signals like leptin and insulin (Thaler et al., 2012; Cai and Liu, 2011). Beyond its effects on energy homeostasis, hypothalamic inflammation impacts other neuroendocrine functions regulated by this brain region (Cai and Liu, 2011). Inflammatory cytokines modulate the activity of the hypothalamic-pituitary-adrenal (HPA) axis, typically enhancing glucocorticoid secretion, which further influences metabolism and immune function (Cai and Liu, 2011; Rivest, 2009). Hypothalamic inflammation also perturbs the regulation of the autonomic nervous system, altering sympathetic and parasympathetic outflow to peripheral metabolic tissues, including adipose tissue, liver, and pancreas (Purkayastha et al., 2011; Yi et al., 2010; Kalsbeek et al., 2010). This autonomic dysregulation creates a feed-forward cycle, where neural regulation of peripheral tissues exacerbates metabolic dysfunction and inflammation (Yi et al., 2010; Kalsbeek et al., 2010).

### 5.2 Microglial activation and neuroinflammation beyond the hypothalamus

While the hypothalamus exhibits particular vulnerability to metabolic inflammation, neuroinflammatory processes in obesity extend to multiple brain regions, including those critical for cognitive function, such as the hippocampus and cortex (Kälin et al., 2015; Buckman et al., 2014). Microglia, the resident immune cells of the CNS, play a central role in orchestrating these widespread neuroinflammatory responses to metabolic dysfunction (Wolf et al., 2013). In healthy CNS, microglia exist in a surveillant state characterized by ramified morphology and continuous monitoring of the neural microenvironment (Nimmerjahn et al., 2005). In obesity and related metabolic disorders, microglia transition to activated states, exhibiting morphological changes and enhanced production of inflammatory mediators that are linked to cognitive impairment (Schafer and Stevens, 2013; Baufeld et al., 2016). This microglial activation occurs in response to multiple signals associated with peripheral metabolic dysfunction, including circulating cytokines, saturated fatty acids, hyperglycemia, and endogenous danger signals released from stressed or damaged neural cells (Hotamisligil, 2017; Wes et al., 2016). The microglial response to metabolic inflammation involves significant transcriptional reprogramming, with altered expression of genes involved in immune signaling, phagocytosis, lysosomal function, and metabolism (Gosselin et al., 2017; Masuda et al., 2020). Single-cell RNA sequencing studies have identified distinct microglial activation states, including disease-associated microglia (DAM) signatures originally described in neurodegenerative conditions, providing a framework for interpreting microglial responses in metabolic inflammation (Masuda et al., 2020; Keren-Shaul et al., 2017). These activation states differ from those observed in neurodegenerative diseases like Alzheimer's, suggesting metabolic inflammation induces unique microglial phenotypes (Jordão et al., 2019). Metabolic activation of microglia involves multiple signaling pathways (Stratoulias et al., 2019). TLR4 activation by saturated fatty acids and endogenous DAMPs triggers MyD88-dependent signaling, leading to NF-κB activation and production of pro-inflammatory cytokines (Shi et al., 2006; Rocha et al., 2016). The NLRP3 inflammasome, activated by metabolic signals including ceramides, ATP, and glucose, mediates processing of IL-1β and IL-18, potent inducers of neuroinflammatory responses (Stienstra et al., 2011). Microglial purinergic receptors, particularly P2Y12 and P2X7, respond to extracellular ATP released during neural stress, further promoting microglial activation and migration (Inoue and Tsuda, 2018; Eyo et al., 2014). Additionally, microglia express receptors for numerous adipokines, including leptin, allowing direct sensing of altered adipokine profiles in obesity (Tang et al., 2007). Activated microglia produces inflammatory cytokines (TNF-α, IL-1β, IL-6), chemokines, and reactive oxygen/nitrogen species that impact neuronal function (Hanisch and Kettenmann, 2007; Smith et al., 2012). These inflammatory mediators modulate synaptic transmission through multiple mechanisms, including altered neurotransmitter dynamics, modulation of receptor trafficking and function, and disruption of neuronal excitability (Liu et al., 2017; Yang et al., 2025). Pro-inflammatory cytokines also impair long-term potentiation (LTP), a key cellular mechanism underlying learning and memory, while promoting long-term depression (LTD)(Goshen et al., 2007; Gruol, 2015). Furthermore, chronic microglial activation leads to aberrant synaptic pruning, where excessive elimination of synapses contributes to cognitive impairment (Schafer et al., 2012). Importantly, microglial activation in metabolic inflammation exhibits regional heterogeneity across the CNS (Cope et al., 2018). The hippocampus, a critical structure for memory formation, shows particularly prominent microglial activation in obesity and metabolic disease models, correlating with impaired hippocampal-dependent memory tasks (Cope et al., 2018; Hao et al., 2016). The prefrontal cortex, essential for executive function and decision-making, also exhibits significant microglial inflammatory responses in metabolic disease (Bocarsly et al., 2015), while the amygdala, involved in emotional processing, shows altered microglial activation that may contribute to anxiety-like behaviors observed in obesity (Guillemot-Legris and Muccioli, 2017; Sharma and Fulton, 2013). This regional pattern of microglial activation aligns with the cognitive and behavioral alterations documented in obesity and metabolic syndrome (Dinel et al., 2011).

### 5.3 Astrocytes, oligodendrocytes, and BBB dysfunction: the extended neuroinflammatory network

While microglia represent primary immune effector cells in the CNS, neuroinflammation in metabolic disorders involves a complex network of cellular interactions, including astrocytes, oligodendrocytes, and cerebrovascular cells (Sofroniew, 2014; Verkhratsky and Nedergaard, 2018). These cell types exhibit distinct responses to metabolic inflammation and contribute significantly to neuroinflammatory progression and associated cognitive dysfunction (Liddelow et al., 2017; Naveed et al., 2025). Astrocytes, the most abundant glial cells in the CNS, perform crucial functions in neurotransmitter homeostasis, metabolic support for neurons, synaptic development, and BBB maintenance (Barres, 2008; Khakh and Sofroniew, 2015; Díaz-Castro et al., 2023). In obesity and metabolic disease, astrocytes adopt reactive phenotypes characterized by hypertrophy, increased GFAP expression, and transcriptional reprogramming (Escartin et al., 2021; Firth et al., 2024). Reactive astrocytes in metabolic inflammation produce pro-inflammatory cytokines (IL-6, TNF-α), chemokines (CCL2, CXCL10), and contribute to extracellular matrix remodeling through secretion of matrix metalloproteinases (Zamanian et al., 2012; Sofroniew, 2015; Rosenberg, 2002, 2009). Astrocytes express receptors for numerous metabolic signals, including insulin, leptin, and, to a lesser extent, adiponectin, allowing direct sensing of metabolic perturbations (Heni et al., 2011; Hsuchou et al., 2009; Parimisetty et al., 2016). Astrocytic responses to metabolic inflammation include altered glutamate homeostasis, with changes in the expression and regulation of glutamate transporters (GLT-1, GLAST) potentially leading to excitotoxicity (Fuente-Martín et al., 2012). Impaired astrocytic glucose uptake and lactate production affect neuronal energetics (Bélanger et al., 2011), while disruption of astrocyte-mediated cholesterol metabolism impacts synaptic plasticity (van Deijk et al., 2017). Reactive astrocytes also exhibit altered production of gliotransmitters, which modulate neuronal function and synaptic plasticity (Perea et al., 2009; Bazargani and Attwell, 2016). Oligodendrocytes and their precursors (OPCs) support axonal integrity and energy metabolism, with important implications for white matter integrity (Nave and Werner, 2014; Saab et al., 2016). Obesity and metabolic syndrome are associated with white matter abnormalities, including reduced fractional anisotropy in diffusion tensor imaging and histological evidence of demyelination (Kullmann et al., 2015). OPCs and mature oligodendrocytes are susceptible to inflammatory cytokines, particularly TNF-α and IL-1β, which can induce apoptosis or dysfunction in these cells (Ye and D'Ercole, 1999; Takahashi et al., 2003). Additionally, oxidative stress can impair oligodendrocyte maturation and promote cell death under inflammatory conditions (French et al., 2009; Back et al., 1998; Peferoen et al., 2014). Oligodendrocyte dysfunction manifests as reduced myelin protein expression, altered myelination patterns, and impaired remyelination capacity (Peferoen et al., 2014; Franklin and Ffrench-Constant, 2008). These changes compromise axonal conduction efficiency and neuronal metabolic support provided by oligodendrocytes (Lee et al., 2012). White matter integrity is critical for inter-regional neural communication, and its disruption- particularly under the influence of neuroinflammatory processes in metabolic disorders—can impair network level signaling and ultimately contribute to cognitive decline (Fields, 2015; Debette and Markus, 2010). Blood–brain barrier dysfunction represents another critical component of neuroinflammation in metabolic disorders (Zhao et al., 2015; Feng et al., 2024). The BBB, formed by specialized endothelial cells connected by tight junctions and supported by pericytes and astrocytic end feet, regulates the exchange of molecules and cells between the circulation and CNS (Zhao et al., 2015; Sweeney et al., 2016). In obesity and related metabolic disorders, BBB integrity is compromised through multiple mechanisms (Feng et al., 2024; Salameh et al., 2016):

- Pro-inflammatory cytokines (TNF-α, IL-1β) disrupt tight junction protein expression and organization through NF-κB-dependent mechanisms (Abbott et al., 2010; Pan et al., 2011).
- Matrix metalloproteinases, elevated in metabolic inflammation, degrade extracellular matrix components and tight junction proteins (Feng et al., 2024; Yang et al., 2007; Rempe et al., 2016).
- Oxidative stress damages endothelial cells and their junctional complexes (Feng et al., 2024; Freeman and Keller, 2012).
- Hyperglycemia induces endothelial dysfunction through formation of AGEs and increased oxidative stress (Prasad et al., 2014; Bogush et al., 2017).
- Dyslipidemia, particularly elevated LDL and oxidized lipoproteins, impairs endothelial function and tight junction integrity (Feng et al., 2024; Zhan et al., 2018).

BBB disruption facilitates increased infiltration of peripheral immune cells and inflammatory mediators into the CNS, amplifying neuroinflammatory processes (Daneman and Prat, 2015; Zenaro et al., 2017). Additionally, compromised BBB function alters the transport of nutrients, including glucose and amino acids, potentially affecting neuronal metabolism (Campos-Bedolla et al., 2014). The regional heterogeneity of BBB dysfunction in metabolic disorders, with particular vulnerability in hypothalamic and hippocampal regions, contributes to the specific pattern of neurological deficits observed in these conditions (Feng et al., 2024; Davidson et al., 2012). Thus, BBB disruption represents a critical interface through which chronic systemic inflammation, dyslipidemia, and hyperglycemia in metabolic disease are transduced into central neuroinflammatory responses and synaptic dysfunction.

### 5.4 Neuroinflammatory impact on synaptic function and cognitive processes

The neuroinflammatory processes triggered by metabolic dysfunction culminate in synaptic alterations that underlie cognitive impairment (Stranahan et al., 2008; Cisternas et al., 2015). Understanding the mechanisms linking neuroinflammation to synaptic dysfunction provides insight into the cognitive deficits observed in obesity and metabolic syndromes and identifies potential therapeutic targets (Guillemot-Legris and Muccioli, 2017; Stranahan et al., 2008). Pro-inflammatory cytokines produced during metabolic inflammation directly modulate synaptic function through multiple mechanisms (Hao et al., 2016; Pan et al., 2023). TNF-α increases surface expression of AMPA receptors while decreasing GABA receptor expression, shifting the excitatory-inhibitory balance toward excitation (Stellwagen and Malenka, 2006; Pribiag and Stellwagen, 2013). IL-1β impairs NMDA receptor-dependent LTP while enhancing NMDA receptor-dependent excitotoxicity through increased receptor phosphorylation (Tong et al., 2012; Viviani et al., 2003). IL-6 modulates neurotransmitter release and receptor function in a context-dependent manner, with predominantly detrimental effects on hippocampal synaptic plasticity under chronic inflammatory conditions (Erta et al., 2012; Tancredi et al., 2000). Microglial phagocytosis of synaptic elements, a process critical for normal circuit refinement during development, becomes dysregulated in neuroinflammation (Schafer and Stevens, 2010). Activated microglia increase expression of complement proteins and their receptors, particularly C1q and CR3, which tag synapses for elimination (Stevens et al., 2007; Stephan et al., 2012). In metabolic disorders such as obesity, inflammatory signaling can also impair adult neurogenesis, particularly in the hippocampal dentate gyrus, a process important for certain forms of learning and memory (Chesnokova et al., 2016).

Pro-inflammatory cytokines and activated microglia can disrupt neural progenitor cell proliferation, survival, and differentiation into mature neurons, potentially contributing to deficits in pattern separation, cognitive flexibility, and memory consolidation observed in metabolic disorders (Chesnokova et al., 2016). At the molecular level, neuroinflammation interferes with signaling pathways essential for synaptic plasticity (Tong et al., 2012; Zhao et al., 2025). Inflammatory activation of JNK and p38 MAPK pathways inhibits insulin and BDNF signaling through their respective receptors, impairing activation of downstream pathways crucial for synaptic plasticity, including PI3K-Akt and ERK1/2 (Tong et al., 2012). Obesity-related inflammation and endocrine disruption also downregulate neurotrophic factors, including BDNF and IGF-1, which are critical for hippocampal neurogenesis, synaptic maintenance, and resilience to metabolic and oxidative stress. Reduced neurotrophic support likely interacts with cytokine-mediated synaptic dysfunction to amplify vulnerability to cognitive decline (Huang and Reichardt, 2003). Inflammation-induced ER stress activates the PKR-like ER kinase (PERK) pathway, leading to phosphorylation of eukaryotic initiation factor 2α (eIF2α) and inhibition of protein synthesis required for long-term synaptic plasticity and memory formation (Ma et al., 2013; Costa-Mattioli and Walter, 2020). The functional consequences of these neuroinflammatory effects manifest as specific cognitive impairments, with deficits in hippocampal-dependent declarative memory and prefrontal cortex-dependent executive functions observed in both humans with obesity and corresponding animal models (Cisternas et al., 2019; Wang et al., 2016). Spatial and recognition memory, assessed through maze tasks and novel object recognition tests in rodent models, show consistent impairment correlating with hippocampal inflammatory markers (Lewis et al., 2019). Executive functions, including cognitive flexibility, response inhibition, and working memory, are similarly compromised, with deficits linked to prefrontal cortical inflammation, and these cognitive impairments associated with metabolic inflammation can be detected even before overt neurodegenerative pathology (Nguyen et al., 2014; Yang et al., 2020). Beyond region-specific changes, obesity and metabolic dysfunction are associated with alterations in large-scale brain networks, including the default mode network, hippocampal-prefrontal circuitry, and fronto-striatal circuits. Functional and structural connectivity studies show disrupted integration within these networks that parallels deficits in memory, executive function, and reward-related decision-making, providing a bridge between cellular neuroinflammatory mechanisms and clinical cognitive manifestations. This suggests potential reversibility, particularly in early stages, through interventions targeting metabolic dysfunction and neuroinflammation (Figure 1).

## 6 Endocrine disruption in metabolic disorders: impact on neurological function

### 6.1 HPA axis dysregulation: stress, metabolism, and neuroinflammation

**Figure 2:**
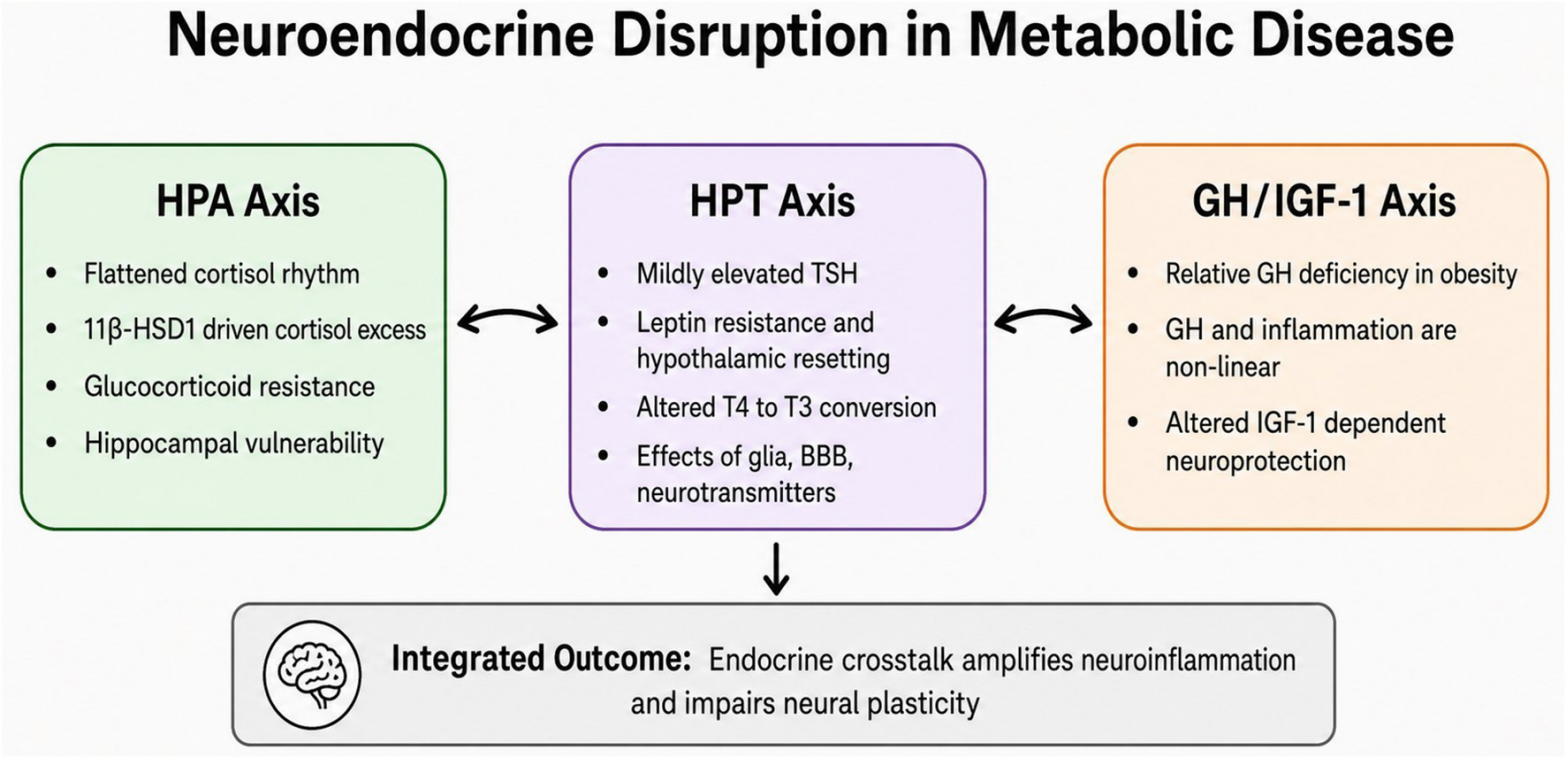
Neuroendocrine disruption in metabolic disease. 11β-HSD1, 11β-hydroxysteroid dehydrogenase type 1; TSH, thyroid-stimulating hormone; BBB, blood–brain barrier; GH, growth hormone; IGF-1, insulin-like growth factor 1.

The hypothalamic–pituitary–adrenal (HPA) axis is a core neuroendocrine system that coordinates stress adaptation, energy balance, and immune regulation. Bidirectional interactions between HPA axis function and metabolic status significantly influence neuroinflammation and cognitive function in obesity and MASLD (Sampada et al., 2025; Shea et al., 2024). HPA activation begins with corticotropin-releasing hormone (CRH) release from the hypothalamic paraventricular nucleus, followed by adrenocorticotropic hormone (ACTH) secretion from the anterior pituitary and glucocorticoid production by the adrenal cortex. Under physiological conditions, glucocorticoids restore homeostasis through broad metabolic actions and negative feedback at hypothalamic and pituitary levels (Tasker and Herman, 2011). Obesity is not typically associated with overt hypercortisolemia, but rather with subtle and biologically meaningful alterations in glucocorticoid dynamics. These include increased basal cortisol production, enhanced local cortisol regeneration in metabolically active tissues, and disturbed circadian rhythmicity. Visceral adiposity particularly correlates with increased 11β-hydroxysteroid dehydrogenase type 1 (11β-HSD1) activity, converting inactive cortisone to active cortisol within adipose tissue, creating tissue-specific hypercortisolism despite normal circulating levels (Seckl and Walker, 2001; Hardy et al., 2021; Lonard et al., 2007). HPA axis dysregulation in obesity manifests as: (1) flattened diurnal cortisol rhythms with impaired morning surge; (2) enhanced cortisol responses to physiological and psychological stimuli; (3) impaired negative feedback sensitivity; and (4) altered peripheral glucocorticoid metabolism. These alterations contribute to functional hypercortisolism in key tissues despite potentially normal circulating levels (Seckl and Walker, 2001; Clarke et al., 2024). Chronically elevated glucocorticoid signaling initially exerts anti-inflammatory effects but eventually induces glucocorticoid resistance through receptor downregulation and altered post-receptor signaling. This phenomenon occurs in both peripheral immune cells and CNS microglia, resulting in enhanced pro-inflammatory cytokine production despite elevated glucocorticoid levels. Chronic glucocorticoid exposure primes microglia and enhances their pro-inflammatory responses to subsequent stimuli, particularly in the hippocampus, a region characterized by high glucocorticoid receptor density (Frank et al., 2014; Calcia et al., 2016). Within the CNS, the hippocampus exhibits vulnerability to HPA axis dysregulation in obesity. Chronic glucocorticoid exposure impairs hippocampal neurogenesis, induces dendritic atrophy, and reduces synaptic plasticity—effects exacerbated by concurrent neuroinflammation. The hippocampus appears especially vulnerable to chronic HPA axis disruption in metabolic disease. Sustained glucocorticoid exposure impairs adult neurogenesis, promotes dendritic remodeling, and weakens synaptic plasticity, and these effects are amplified in the presence of concurrent neuroinflammation. Together, these mechanisms are likely to contribute to the deficits in hippocampal-dependent learning and memory observed in obesity and MASLD. Experimental studies further suggest that interventions that normalize HPA function or reduce inflammatory signaling can partially restore hippocampal neurogenesis and improve cognitive performance (Lucassen et al., 2015; Sung et al., 2020). HPA dysregulation also interacts with behavior and circadian biology in ways that perpetuate metabolic disease. Disturbed glucocorticoid signaling contributes to sleep fragmentation, altered circadian timing, stress-related feeding, and increased preference for calorie-dense foods. These downstream effects reinforce obesity, systemic inflammation, and maladaptive neuroendocrine signaling, creating a self-sustaining feed-forward loop between stress biology, metabolic dysfunction, and impaired brain health (Figure 2) (Dallman et al., 2003; Sominsky and Spencer, 2014).

### 6.2 Hypothalamic–pituitary–thyroid axis disruption in metabolic disorders

The hypothalamic–pituitary–thyroid (HPT) axis regulates energy expenditure, metabolism, and neural development through thyroid hormone production and release. This axis encompasses thyrotropin-releasing hormone (TRH) neurons in the hypothalamic paraventricular nucleus, thyroid-stimulating hormone (TSH) producing cells in the anterior pituitary, and the thyroid gland secreting thyroxine (T4) and triiodothyronine (T3). Thyroid hormones influence multiple aspects of neural function, including neurogenesis, myelination, synaptic plasticity, and cerebrovascular regulation (Williams, 2008).

Obesity and MASLD are associated with subtle yet clinically relevant HPT axis alterations, typically manifesting as modestly elevated TSH levels with normal or elevated free T3 and typically normal free T4. This pattern differs from primary hypothyroidism, suggesting complex adaptations rather than simple thyroid dysfunction. Contributing mechanisms include:

- Hypothalamic inflammation affecting TRH neuronal function, altering the set point for negative feedback regulation by thyroid hormones, with JNK signaling implicated in this dysregulation (Zhang et al., 2008; De Souza et al., 2005).
- Leptin resistance disrupting normal leptin-mediated stimulation of TRH expression in the paraventricular nucleus (Légrádi et al., 1997; Fekete and Lechan, 2014).
- Altered peripheral thyroid hormone metabolism in obesity by increased conversion of T4 to T3, partly mediated by enhanced deiodinase activity in adipose tissue, particularly within expanded fat depots (Sanyal and Raychaudhuri, 2016; Longhi and Radetti, 2013).
- Reduced tissue sensitivity to thyroid hormones in obesity, analogous to insulin resistance, resulting in compensatory increases in circulating hormone levels (Sanyal and Raychaudhuri, 2016; Longhi and Radetti, 2013).

The relationship between HPT axis alterations and neuroinflammation involves multiple pathways. Thyroid hormones modulate microglial and astrocyte function, with both hypo- and hyperthyroidism capable of inducing glial activation and inflammatory responses. TSH receptors expressed on immune cells and adipocytes allow elevated TSH to stimulate pro-inflammatory cytokine production, particularly IL-6, through cAMP-protein kinase A signaling. Furthermore, TSH inhibits adipocyte triglyceride lipase activity, potentially exacerbating adipose dysfunction and associated inflammation (Longhi and Radetti, 2013; Jiang et al., 2015). HPT axis dysregulation impacts neural function through several mechanisms relevant to cognitive performance:

- Altered neural energy metabolism, characterized by changes in mitochondrial function and impaired glucose utilization in the brain (Camandola and Mattson, 2017).
- Disruption of key neurotransmitter systems, including acetylcholine, serotonin, dopamine, and glutamate signaling pathways (Teleanu et al., 2022).
- Impaired neurotrophic support, particularly reduced brain-derived neurotrophic factor (BDNF) expression, compromises neuronal survival and synaptic plasticity (Park and Poo, 2013).
- Cerebrovascular dysfunction and increased blood–brain barrier permeability, facilitating the entry of peripheral inflammatory mediators into the central nervous system (Varatharaj and Galea, 2017).

Clinically, these thyroid-related changes are increasingly viewed as secondary to obesity rather than evidence of intrinsic thyroid gland disease. This interpretation is supported by studies showing that weight loss and metabolic improvement are often accompanied by normalization of TSH and related thyroid parameters. Accordingly, recognition of HPT axis remodeling in obesity may be more informative for understanding neuro-metabolic vulnerability than for diagnosing primary thyroid dysfunction (Figure 2) (Longhi and Radetti, 2013; Reinehr, 2010).

### 6.3 Growth hormone/IGF-1 axis alterations in metabolic dysfunction

The growth hormone (GH)/insulin-like growth factor-1 (IGF-1) axis is another neuroendocrine system profoundly altered in obesity, with significant implications for neuroinflammation and cognitive function. The GH–IGF-1 axis is characterized by pulsatile GH secretion from the anterior pituitary under the regulation of hypothalamic growth hormone-releasing hormone (GHRH) and somatostatin, which in turn stimulates IGF-1 production primarily in the liver, but also in peripheral tissues and the CNS (Clemmons, 2007). Obesity is consistently associated with relative GH deficiency, reduced pulse amplitude, frequency, and 24-h secretion resulting from: (1) impaired hypothalamic GHRH release; (2) enhanced somatostatin tone; (3) reduced pituitary GHRH responsiveness; and (4) accelerated GH clearance. Interestingly, IGF-1 levels are often normal or only modestly reduced despite decreased GH secretion, suggesting a state of hepatic GH resistance with relative preservation of IGF-1 production, a pattern that contrasts with conditions such as anorexia nervosa (Møller and Jørgensen, 2009; Misra and Klibanski, 2014). The GH/IGF-1 axis significantly regulates inflammatory processes in multiple tissues including the brain. Both excess and deficiency of GH/IGF-1 signaling can promote inflammatory responses through different mechanisms. GH excess directly enhances hypothalamic inflammation independent of IGF-1 through JAK2-STAT5 and other pathways that activate inflammatory transcription factors including NF-κB. This GH axis dysregulation has been linked to hypothalamic neuroinflammation and insulin resistance, potentially contributing to a self-perpetuating cycle of metabolic dysfunction and inflammatory signaling (De Souza et al., 2005; Kullmann et al., 2016). Paradoxically, GH deficiency or resistance, as observed in obesity, also promotes inflammation by decreasing production of anti-inflammatory mediators and impairing resolution of inflammatory responses. This suggests a U-shaped relationship between GH signaling and inflammation, whereby both excess and deficiency are linked to increased neuroinflammatory activity (Møller and Jørgensen, 2009; Bartke, 2008). IGF-1 exerts largely anti-inflammatory effects in the CNS, including suppression of pro-inflammatory cytokine production and promotion of anti-inflammatory mediator expression. It also enhances microglial phagocytosis of cellular debris and supports neuronal survival and synaptic plasticity, thereby partially counteracting the detrimental effects of neuroinflammation (Labandeira-Garcia et al., 2017; Dyer et al., 2016; Fernandez and Torres-Alemán, 2012). The GH/IGF-1 axis also influences neuroinflammation indirectly through modulation of insulin sensitivity. GH promotes insulin resistance by enhancing lipolysis and increasing circulating free fatty acids, whereas IGF-1 enhances insulin sensitivity through shared signaling pathways, including activation of the PI3K–Akt pathway (Clemmons, 2007; Møller and Jørgensen, 2009). Insights from aging and longevity research further highlight the complex relationships between GH/IGF-1 signaling, inflammation, and cognition. GH receptor-deficient mice exhibit extended lifespan, reduced age-related neuroinflammation, and preserved cognitive function compared to wild-type controls. However, severe GH/IGF-1 deficiency during development adversely affects brain structure and function, underscoring the importance of appropriate signaling during critical neurodevelopmental windows (Bartke, 2019; Zhou et al., 2022). Therapeutic targeting of the GH/IGF-1 axis in metabolic disorders has produced mixed results. GH administration in GH-deficient states may reduce certain inflammatory markers but can also exacerbate insulin resistance (Figure 2). In contrast, IGF-1 administration demonstrates more consistent anti-inflammatory effects, although concerns remain regarding its potential mitogenic properties (Clemmons, 2007; Møller and Jørgensen, 2009).

### 6.4 Integration of neuroendocrine signals in metabolic brain disorders

The HPA, HPT, and GH/IGF-1 axes operate as an integrated neuroendocrine network with extensive crosstalk and mutual regulation. Glucocorticoids modulate both HPT and GH/IGF-1 function through multiple mechanisms, including inhibition of TRH expression, suppression of TSH release, alterations in peripheral thyroid hormone metabolism, inhibition of GH secretion, reduced hepatic GH receptor expression, and modulation of tissue-specific IGF-1 production (Fekete and Lechan, 2014; Møller and Jørgensen, 2009). Thyroid hormones reciprocally modulate HPA axis function, with hypothyroidism generally associated with enhanced and hyperthyroidism with reduced HPA activity, mediated through effects on hypothalamic corticotropin-releasing hormone (CRH) neurons, anterior pituitary corticotrophs, and adrenal cortical cells. Thyroid hormones also influence GH secretion and peripheral IGF-1 responsiveness, with hypothyroidism typically reducing and hyperthyroidism enhancing GH pulsatility (Fekete and Lechan, 2014; Sanyal and Raychaudhuri, 2016). GH and IGF-1 similarly interact with both the HPA and HPT axes. GH excess is associated with enhanced peripheral cortisol metabolism, while IGF-1 modulates hypothalamic–pituitary sensitivity to glucocorticoid negative feedback. The GH/IGF-1 axis also influences thyroid function, with GH therapy typically increasing peripheral conversion of T4 to T3 (Sanyal and Raychaudhuri, 2016; Møller and Jørgensen, 2009). These neuroendocrine interactions occur against a background of obesity-associated neuroinflammation, giving rise to complex feedback loops. Pro-inflammatory cytokines can activate the HPA axis while suppressing HPT and GH/IGF-1 signaling, and chronic inflammation is associated with tissue-specific hormone resistance, further complicating the neuroendocrine profile in metabolic disorders (Hotamisligil, 2006; Tsigos and Chrousos, 2002; Warner and Beckett, 2010). The integrated function of these systems has significant effects on cognitive processes. Glucocorticoids, thyroid hormones, and IGF-1 influence hippocampal neurogenesis, synaptogenesis, and synaptic plasticity which are key processes underlying learning and memory. In addition, these hormones modulate neurotransmitter systems and contribute to the regulation of cerebral glucose metabolism, cerebral blood flow, and blood–brain barrier function (McEwen and Morrison, 2013; Aguiar-Oliveira et al., 2026; Banks, 2016). The clinical implications include the recognition that isolated measurement of single hormones may inadequately capture the complex endocrine dysregulation underlying obesity-associated cognitive impairment. Therapeutic approaches targeting multiple neuroendocrine pathways may therefore be more effective than narrowly focused interventions. Inter-individual variability in neuroendocrine responses to obesity and weight loss may contribute to the heterogeneity in cognitive outcomes observed in metabolic disorders. In addition, sex hormones modulate many of the neuro-immune-endocrine pathways discussed above, contributing to sex differences in the prevalence and clinical course of MASLD and metabolic-associated cognitive impairment. Estrogen exerts anti-inflammatory, vasoprotective, and metabolic effects that may attenuate neuroinflammation and cognitive risk in premenopausal women, whereas its decline after menopause parallels rising rates of MASLD, visceral adiposity, and dementia. In men, androgen levels and fat distribution patterns interact with liver and adipose inflammation in ways that may confer higher risk at younger ages, highlighting the need for sex-specific mechanistic and clinical studies (Ryoo et al., 2024; Kullmann et al., 2012).

## 7 Therapeutic implications and future directions

### 7.1 Targeting neuroinflammation in metabolic brain disorders

**Figure 3:**
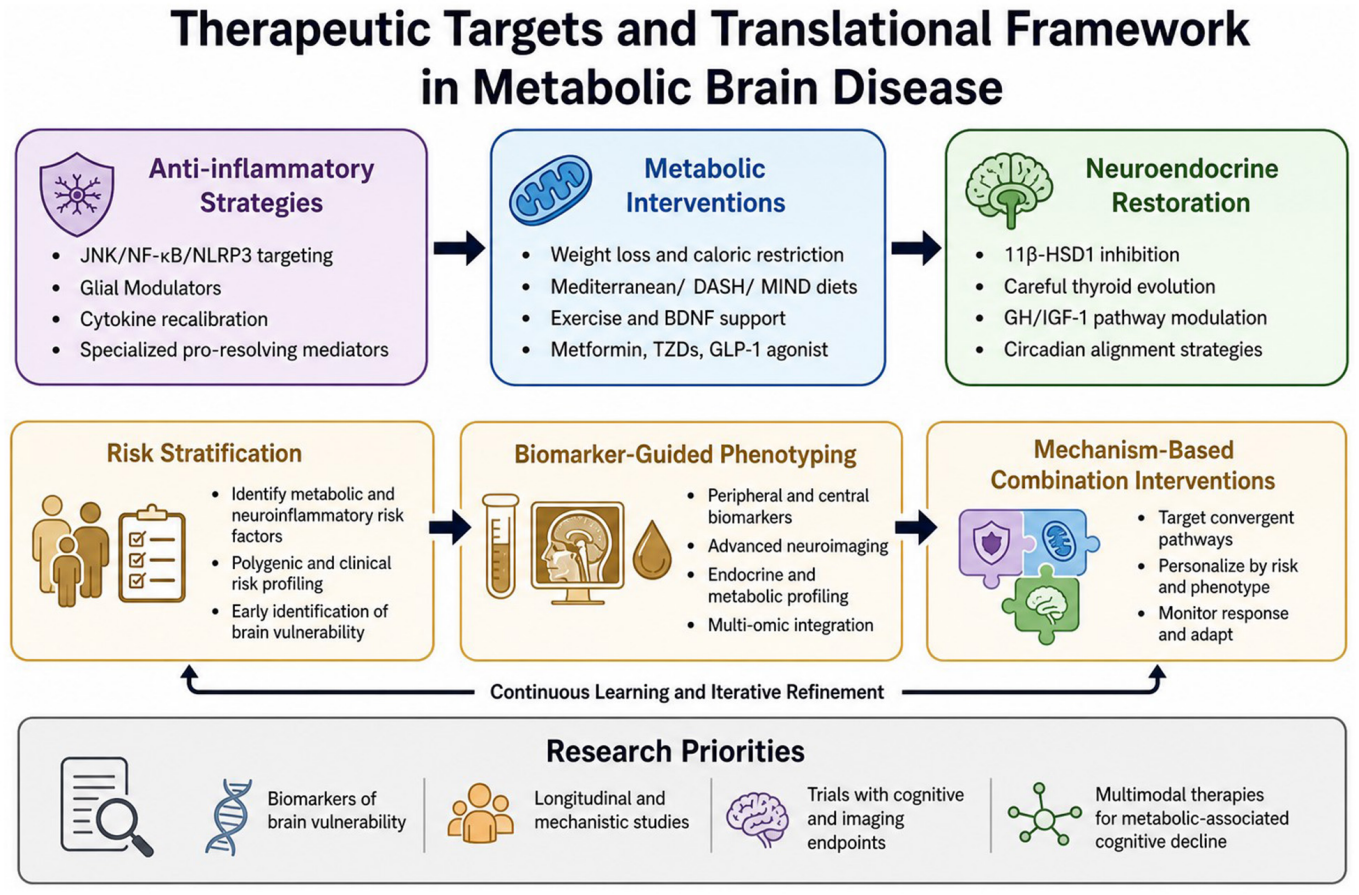
Therapeutic targets and translational framework in metabolic brain disease. JNK, c-Jun N-terminal kinase; NF-κB, nuclear factor kappa-light-chain-enhancer of activated B cells; NLRP3, NLR family pyrin domain containing 3; DASH, dietary approaches to stop hypertension; MIND, Mediterranean-DASH intervention for neurodegenerative delay; BDNF, brain-derived neurotrophic factor; TZDs, thiazolidinediones; GLP-1, glucagon-like peptide-1; 11β-HSD1, 11β-hydroxysteroid dehydrogenase type 1; G, growth hormone; IGF-1, insulin-like growth factor 1.

Within the proposed neuro-immune-endocrine framework, therapeutic opportunities can be conceptualized along three interacting domains: (1) reducing systemic metabolic and inflammatory load, (2) modulating CNS glial and neuroendocrine responses, and (3) preserving network-level connectivity and cognitive function. Recognition of neuroinflammation as a central mechanistic bridge between metabolic dysfunction and cognitive impairment has motivated the search for targeted anti-inflammatory therapies in metabolic brain disorder. Broad anti-inflammatory approaches such as non-steroidal anti-inflammatory drugs (NSAIDs) have produced inconsistent results in experimental and clinical contexts, and their long-term use is constrained by cardiovascular and gastrointestinal toxicities (Bally et al., 2017). These limitations have shifted attention toward pathway-selective strategies that more precisely interrupt metabolic inflammatory signaling (Figure 3). Pathway-specific inhibitors targeting JNK, IKK/NF-κB, and inflammasome cascades demonstrate superior efficacy in preclinical metabolic disease models. Hypothalamic JNK inhibition has been shown to reduce neuroinflammation and improve leptin and insulin sensitivity in obesity models. Similarly, inhibition of the NLRP3 inflammasome attenuates microglial activation and is associated with improved cognitive function in diet-induced obesity (Sabio and Davis, 2014; Guo et al., 2020). Glial modulators that normalize microglial function without immunosuppression (e.g., minocycline, GLP-1 receptor agonists) reduce pro-inflammatory cytokine production while enhancing phagocytic capacity and neurotrophic factor expression. Therapeutic strategies targeting astrocytic neuroprotective functions or oligodendrocyte resilience may offer complementary benefits by addressing multiple aspects of glial dysfunction (Liddelow and Barres, 2017). Cytokine-specific interventions, including IL-1 receptor antagonism with anakinra and TNF-α inhibition with etanercept, have shown efficacy in experimental models. However, the pleiotropic roles of cytokines in neural function necessitate more nuanced modulatory approaches to achieve optimal outcomes (Dinarello et al., 2012; McCoy and Tansey, 2008; Beurel et al., 2020). Specialized pro-resolving mediators (SPMs)—including resolvins, protectins, and maresins—represent an emerging therapeutic paradigm facilitating transition from inflammation to tissue repair. Resolvin D1 administration has been shown in some cases to reduce hypothalamic inflammation and improve leptin sensitivity and metabolic function in obesity models, potentially contributing to the resolution of chronic neuroinflammation characteristic of metabolic disorders (Serhan, 2014; González-Périz et al., 2009).

### 7.2 Metabolic interventions with neurological benefits

Because neuroinflammation arises in large part from systemic metabolic dysfunction, interventions that improve metabolic health remain foundational. Caloric restriction, sustained weight loss, and improved insulin sensitivity are associated with reductions in peripheral and central inflammation, partial normalization of neuroendocrine signaling, and measurable improvements in cognition (Blüher, 2019; Cabo and Mattson, 2019; Veronese et al., 2017). These effects reinforce the principle that therapies directed at the metabolic source of inflammatory signaling may yield broad neurological benefit.

Specific dietary patterns confer particular neuroinflammatory benefits beyond caloric restriction. Mediterranean, DASH (Dietary Approaches to Stop Hypertension), and MIND (Mediterranean-DASH Intervention for Neurodegenerative Delay) diets associate with reduced inflammation and enhanced cognitive outcomes. Omega-3 polyunsaturated fatty acids serve as SPM precursors while exhibiting direct anti-inflammatory properties. Dietary polyphenols have been shown to exert antioxidant and anti-inflammatory effects, with compounds such as resveratrol and curcumin demonstrating potential for reducing neuroinflammation (Vauzour et al., 2010; Heneka et al., 2015; Albadrani et al., 2024).

Exercise provides multifaceted benefits through reduced systemic inflammation, enhanced insulin sensitivity, improved cerebral perfusion, and increased neurotrophic factor production, particularly BDNF. These physiological alterations are associated with improvements in cognitive function, particularly in domains such as executive function and memory, and may occur even in the absence of significant weight reduction (Smith et al., 2010; Chávez-Manzanera et al., 2022).

Several metabolic drugs also show promise for neurological benefit. Metformin may reduce neuroinflammation through AMPK activation, improved metabolic control, and attenuation of oxidative stress. Thiazolidinediones act through PPAR-γ signaling to suppress inflammatory pathways, while GLP-1 receptor agonists have emerged as particularly compelling because they may reduce microglial activation, enhance autophagy, and promote synaptic and neurotrophic resilience (Monti et al., 2022; Hölscher, 2022).

Recent advances in MASLD/MASH therapeutics, including agents targeting fibrosis and metabolic dysregulation, have expanded options for liver-directed treatment, but to date no clinical trials have definitively demonstrated that MASH-targeted therapies improve cognitive outcomes. This gap highlights a key opportunity for future interventional studies that incorporate standardized cognitive endpoints and neuroimaging measures alongside hepatic and metabolic outcomes. Collectively, these observations support a treatment framework in which metabolic therapies are viewed not only as cardiometabolic interventions but also as potential brain-protective strategies (Figure 3).

### 7.3 Neuroendocrine modulation to restore cognitive function

Interventions targeting specific hormone systems represent promising therapeutic approaches given the profound neuroendocrine disruption in metabolic disorders. Inhibitors of 11β-HSD1, which reduce local cortisol regeneration, have shown cognitive benefits in preclinical models and early clinical studies, and may be particularly relevant in visceral obesity, where tissue-specific glucocorticoid activity is increased despite normal circulating levels (Sandeep et al., 2004; Rask et al., 2001; Stimson and Walker, 2013). Thyroid hormone modulation requires careful consideration of systemic effects. While overt hypothyroidism necessitates appropriate replacement, approaches to subtle thyroid alterations in obesity remain undefined. Observational studies suggest that normalization of elevated TSH accompanying obesity can parallel improvements in metabolic parameters and occasionally cognitive measures, but causality remains uncertain and there is insufficient evidence to recommend thyroid hormone therapy solely to treat mild TSH elevation for cognitive benefit. Any consideration of thyroid modulation must weigh uncertain neurocognitive gains against potential cardiovascular and skeletal risks (Samuels, 2008; Sepúlveda et al., 2024). The GH/IGF-1 axis presents a particular complexity for therapeutic intervention. While GH replacement has been shown to improve cognitive function in states of deficiency, the relative GH deficiency observed in obesity occurs within a distinct metabolic context that may limit or complicate the effects of exogenous GH administration. Alternative approaches include ghrelin agonists, which stimulate endogenous GH secretion while exerting additional anti-inflammatory effects, and IGF-1 or IGF-1 mimetics, which may provide neuroprotective benefits with fewer diabetogenic effects (Müller et al., 2015; Zhang et al., 2025; Vijayakumar et al., 2011). Interventions addressing integrated neuro–immune–endocrine function may prove most efficacious. Circadian rhythm normalization through behavioral approaches (consistent sleep-wake scheduling, timed feeding, morning light exposure) and chronobiological agents targeting clock mechanisms demonstrate preliminary metabolic and cognitive benefits (Figure 3).

### 7.4 Toward risk stratification and mechanism-based interventions

Given the heterogeneity of metabolic-associated cognitive impairment, a clinically useful translational framework should incorporate risk stratification, mechanism-based phenotyping, and multi-target intervention design. A conceptual risk-stratification approach might integrate metabolic status (e.g., obesity class, insulin resistance, HbA1c), MASLD stage and fibrosis indices, inflammatory markers (CRP, IL-6), endocrine profiles (cortisol rhythm, TSH, IGF-1), vascular risk factors, and baseline cognitive assessments to identify individuals at higher risk of accelerated cognitive decline.

Because different patients may be driven by distinct dominant mechanisms, such as inflammatory, vascular, or endocrine dysregulation, mechanism-based endophenotyping using a limited biomarker set (for example, CRP/IL-6, cortisol rhythm, TSH, IGF-1, liver stiffness scores) could guide individualized therapeutic strategies. In this framework, lifestyle interventions provide a foundational modality that simultaneously improves adiposity, insulin sensitivity, inflammatory tone, and circadian alignment, upon which targeted pharmacologic or neuroendocrine therapies can be layered to address residual dominant pathways—such as GLP-1 receptor agonists or metformin for persistent insulin resistance, anti-inflammatory agents for ongoing low-grade inflammation, or circadian and stress-management interventions for HPA axis abnormalities.

## 8 Conclusions and future perspectives

The multifaceted interactions between metabolic dysfunction, neuroinflammation, and neuroendocrine perturbations in obesity and MASLD constitute a pivotal domain for understanding the neurological sequelae of metabolic disorders. This synthesis of contemporary evidence yields several salient paradigms: (1) neuroinflammatory processes serve as primary mediators linking metabolic aberrations to cognitive deterioration, with microglia functioning as central cellular effectors; (2) CNS inflammation exhibits topographical specificity, with hypothalamic vulnerability representing a critical nexus between peripheral metabolism and neural function; (3) principal neuroendocrine axes undergo substantial dysregulation in obesity, both contributing to and being modulated by inflammatory cascades; and (4) metabolic-associated cognitive impairment likely follows a progressive trajectory in which early, predominantly functional changes may be at least partially modifiable with effective metabolic and lifestyle interventions, whereas later stages may reflect more fixed structural damage. Despite significant advances, critical knowledge gaps persist. Future investigations should prioritize: precise characterization of region-specific glial phenotypes utilizing single-cell technologies; elucidation of the temporal progression of neuroinflammatory changes to identify intervention windows; examination of sexual dimorphism in neuro–immune–endocrine responses; development of sensitive biomarkers for identifying at-risk individuals; and implementation of appropriately designed clinical trials with cognitive endpoints following metabolic interventions. Overall, the concept of metabolic-associated cognitive decline provides a useful integrative framework for future investigation. It emphasizes that brain dysfunction in obesity and MASLD arises from convergent systemic and central mechanisms and that these mechanisms are, at least in part, biologically modifiable. Available human data indicate that weight loss, dietary change, and physical activity can improve subjective cognition and some cognitive test scores in obesity and diabetes, but it remains uncertain to what extent such interventions alter long-term neurodegenerative risk trajectories. Advancing this field will require coordinated translational work spanning metabolism, neuroscience, immunology, and endocrinology, with the goal of developing preventive and therapeutic strategies that protect brain health in the setting of metabolic disease.
